# Evaluating CMAQ Representation of Wildfire PM_2·5_ in California (2008–2018): Regime-Dependent Performance and Nonlinear Error Structure

**DOI:** 10.21203/rs.3.rs-11167409/v1

**Published:** 2026-11-01

**Authors:** Eimy X. Bonilla, Jose Perez-Chavez, Daja Elum, Olutoyosi Oyelowo, Samuel Kane, Rachel E. Connolly, Miriam E. Marlier, Jihoon Jung, Mohammad Al-Hamdan, Claire L. Schollaert, John C. Flunker, Huikyo Lee, Sambit Bhattacharya, Osinachi Ajoku, Christopher Blaszczak-Boxe, Joseph L. Wilkins

**Affiliations:** Howard UniversityDepartment of Earth, Environment, and Equity, Graduate Program in Atmospheric Sciences; Howard UniversityDepartment of Earth, Environment, and Equity, Graduate Program in Atmospheric Sciences; Howard UniversityDepartment of Earth, Environment, and Equity, Graduate Program in Atmospheric Sciences; Howard UniversityDepartment of Earth, Environment, and Equity, Graduate Program in Atmospheric Sciences; Howard UniversityDepartment of Earth, Environment, and Equity, Graduate Program in Atmospheric Sciences; University of California, Los Angeles; University of California, Los Angeles; University of North Carolina at Chapel Hill; University of Mississippi; University of California, Los Angeles; University of Iowa; Jet Propulsion Laboratory; Fayetteville State University; Howard UniversityDepartment of Earth, Environment, and Equity, Graduate Program in Atmospheric Sciences; Howard UniversityDepartment of Earth, Environment, and Equity, Graduate Program in Atmospheric Sciences; Howard UniversityDepartment of Earth, Environment, and Equity, Graduate Program in Atmospheric Sciences

**Keywords:** wildfire PM_2·5_, CMAQ model evaluation, air quality modeling, fire emissions, atmospheric transport

## Abstract

We evaluated daily paired Community Multiscale Air Quality (CMAQ) estimates and observations during California fire seasons (June–October), 2008–2018, using conventional metrics, classifications at 12 and 35 μg m^−3^, two case studies, and a leakage-resistant Random Forest diagnostic. After quarantining 18 CMAQ total PM_2·5_ values above 557 μg m^−3^ without capping, the primary analysis contained 187,051 records. Observed and modeled means were 10.69 and 9.29 μg m^−3^; Pearson r was 0.502 (r^2^ = 0.252), mean bias was − 1.40 μg m^−3^, and RMSE was 9.96 μg m^−3^. CMAQ detected 45.4% of observed events at the descriptive/former annual level of 12 μg m^−3^ and 40.8% at the current 35 μg m^−3^ 24-hour standard level. At 35 μg m^−3^, CMAQ produced more events overall but missed 1,273 of 2,151 observed events because modeled and observed events often did not coincide by site-day. The Random Forest excluded observed PM_2·5_, CMAQ total PM_2·5_, and the no-fire component from its primary features. In an untouched site-year test partition (n = 37,430), R^2^ was 0.711, RMSE was 5.82 μg m^−3^, and MAE was 3.44 μg m^−3^; fire-only CMAQ PM_2·5_ was the dominant predictor. CMAQ remains useful for regional and multi-year analyses, but paired-event detection and localized extreme-smoke estimates require explicit uncertainty treatment.

## 1. Introduction

Wildfire smoke has become an increasingly important determinant of PM_2·5_ in the western United States, offsetting part of the long-term improvement achieved for other emission sources [1, 2]. California is a critical setting because anthropogenic warming has increased wildfire activity and the state combines a large smoke burden with marked geographic and meteorological heterogeneity [3].

Wildfire PM_2·5_ is associated with respiratory, cardiovascular, reproductive, and mortality outcomes [4-7]. Risk is shaped by exposure, baseline health, age, occupation, housing, access to care, and adaptive capacity; race or ethnicity alone is not treated as a biological vulnerability attribute [8-11].

Chemical transport models provide spatially complete estimates where monitoring is sparse. The Community Multiscale Air Quality (CMAQ) model is used by the U.S. Environmental Protection Agency and supports source-attribution, exposure, and health analyses, but wildfire emissions, plume injection, transport, and sub-grid gradients remain difficult to represent [12, 13]. In this manuscript, wildfire-attributable PM_2·5_ means the modeled difference between total and no-fire CMAQ simulations; it is not a direct measure of fire intensity or fire radiative power.

CMAQ-derived wildfire PM_2·5_ has been used in health, vulnerability, and economic analyses [11, 14, 15]. Wilkins et al. [13] evaluated wildfire-related CMAQ PM_2·5_ and ozone across the contiguous United States for 2008–2012; the present study extends paired evaluation through 2018 and focuses on California, where an 11-year record, extensive monitoring, and contrasting source and terrain settings permit evaluation across years, concentrations, and individual events.

We combined conventional model-evaluation metrics with three complementary analytical dimensions. Temporal scale refers to full-period and year-specific fire-season summaries; concentration regime refers to background, moderate, and high observed PM_2·5_; and event detection refers to paired site-day classification at specified concentration levels. A grouped, leakage-resistant Random Forest then tested whether nonlinear spatial-temporal and modeled wildfire information explained CMAQ error beyond those summaries.

We evaluated daily CMAQ total PM_2·5_ against monitoring observations for June–October 2008–2018, recalculated conventional descriptive and paired threshold-detection statistics, examined the prespecified 2008 Klamath and 2013 Rim fire windows, and quantified nonlinear error structure with an untouched site-year test partition. The machine-learning analysis is diagnostic: it identifies structured error but does not provide an operational correction factor.

## 2. Data and Methods

### 2.1 Observations and paired dataset

In-situ PM_2·5_ observations were obtained from the U.S. Environmental Protection Agency Air Quality System (AQS), the Interagency Monitoring of Protected Visual Environments (IMPROVE), and the Clean Air Status and Trends Network (CASTNET). The monitor distribution is shown in Fig. S1. The principal analyses used daily CMAQ–AQS pairs supplied by the U.S. EPA and processed with the Atmospheric Model Evaluation Tool (AMET) [16]: AMET associated each monitor-day with the CMAQ grid cell containing that monitor. The frozen dataset contained 304 unique AQS site identifiers. Analyses were limited to June–October 2008–2018 to use a consistent California fire-season window. IMPROVE elemental and black carbon measurements provided combustion-related context for the two fire case studies.

Records lacking a finite date, location, observed PM_2·5_, CMAQ total PM_2·5_, or CMAQ no-fire PM_2·5_ value were excluded. Observed concentrations below 0 or above 557 μg m^−3^ were also excluded; zero observations were retained. Each retained record was assigned a monitoring-site identifier and year so observations from the same site-year remained together during machine-learning validation.

### 2.2 CMAQ simulations and wildfire attribution

CMAQ simulations followed the configurations described by Wilkins et al. [13] and Jung et al. [11] and used CMAQ versions 5.0.1–5.3. Meteorological inputs were generated with the Weather Research and Forecasting model at 12-km horizontal resolution and 35 vertical layers extending to 50 hPa; initial and boundary conditions were derived from the North American Mesoscale model. The source files identify simulations with and without wildfire emissions. Prescribed burning and wildfire emissions are represented separately in the CMAQ inputs, whereas agricultural burning is not sufficiently delineated in the paired data. The analysis therefore does not infer agricultural-burning effects.

Two CMAQ fields were evaluated: total PM_2·5_ from all modeled sources and a no-fire simulation that excluded wildfire emissions. Wildfire-attributable PM_2·5_ was calculated as CMAQ total minus CMAQ no-fire PM_2·5_. Fig. S2 summarizes its interannual variation.

### 2.3 Quality control and descriptive evaluation

The validity rules yielded 187,069 paired records. Eighteen otherwise-valid records had CMAQ total PM_2·5_ above 557 μg m^−3^. Because their provenance could not be verified against the raw CMAQ output, these values were quarantined rather than capped or altered. The primary analysis therefore contained 187,051 records. A prespecified raw-extreme sensitivity analysis restored all 18 records to quantify their influence.

We used conventional model-evaluation statistics to summarize observed and modeled means and calculated Pearson correlation (r), its square (r^2^), mean bias (CMAQ minus observed), mean absolute error (MAE), root mean square error (RMSE), normalized mean bias (NMB), and normalized mean error (NME). Metrics were calculated for the full fire-season dataset, by year, and by observed concentration regime. Correlation and the coefficient of determination from regression models are reported separately to avoid treating them as interchangeable.

### 2.4 Concentration regimes and threshold detection

Observed PM_2·5_ defined three descriptive regimes: background (< 12 μg m^−3^), moderate (12 to < 35 μg m^−3^), and high (≥ 35 μg m^−3^). The 12 μg m^−3^ boundary is a descriptive cut point retained from the original analysis and the former annual primary standard; it is not a daily regulatory standard. The 35 μg m^−3^ level is the current 24-hour PM_2·5_ standard, although formal attainment is based on the 98th percentile averaged over three years. The current 9 μg m^−3^ annual standard was not applied to daily values or incomplete-year fire-season means [17]. For each level, an observed event occurred when observed PM_2·5_ met or exceeded the level, and a modeled detection occurred when CMAQ total PM_2·5_ did so on the paired site-day. We calculated true positives, false negatives, false positives, true negatives, sensitivity, specificity, precision, balanced accuracy, F1 score, critical success index, and frequency bias.

As a post hoc lag sensitivity analysis, we compared CMAQ on day t with observations at the same monitoring site from t − 3 through t + 3 days. Positive lags indicate that the modeled value preceded the observation. Pearson r, MAE, RMSE, and F1 scores at 12 and 35 μg m^−3^ were recalculated using the available site-date pairs. This analysis tests whole-day offsets, not subdaily plume timing.

### 2.5 Fire case studies

The 2008 Klamath and 2013 Rim fire windows were prespecified in the source dataset and retained because they provide geographically and temporally distinct events with complete paired records. Monitors within 200 km of the recorded fire origin were included. The radius is geometric and does not account for wind direction; this limits plume-specific attribution. Descriptive statistics were recalculated from the same quality-controlled daily pairs as the primary analysis. CMAQ fire-only PM_2·5_ was compared with IMPROVE carbonaceous aerosol measurements to assess whether simulated wildfire variability tracked combustion-related observations. Spatial evolution and correlation diagnostics are provided in Figs. S5–S8, and case-study metrics are summarized in Table S2.

### 2.6 Leakage-resistant Random Forest diagnostic

We modeled daily CMAQ total PM_2·5_ error, defined as CMAQ total minus observed PM_2·5_, using a Random Forest [18]. Observed PM_2·5_ and CMAQ total PM_2·5_ were excluded from the feature matrix because they directly define the target. CMAQ no-fire PM_2·5_ was also excluded because using it together with fire-only PM_2·5_ would reconstruct CMAQ total PM_2·5_. Retained predictors were CMAQ fire-only PM_2·5_, latitude, longitude, year, month, day of year, geometric distance to the recorded fire origin, a distance-missing indicator, and daily observation count. No fire radiative power, fireline intensity, or other direct fire-intensity variable was available. Observed regime, fire name, and fire-window labels were withheld from training and used only for post hoc evaluation.

A site-year group contained all retained observations from one monitoring site in one calendar year. The 2,139 unique groups were split deterministically: 428 groups (37,430 observations; approximately 20% of groups) were reserved as an untouched test partition, and the remaining 1,711 groups were used for model development. Five-fold GroupKFold cross-validation was conducted within the development partition to quantify out-of-group performance and variability; it was not used for hyperparameter optimization. Missing distance-to-fire values were median-imputed using the relevant training data only, while the missingness indicator was retained. Random Forest hyperparameters were fixed before evaluation rather than selected through grid or randomized search: 160 trees, maximum depth 24, minimum terminal-node size 10, 70% of predictors considered at each split, and random seed 20260905. These settings combined ensemble averaging with explicit constraints on tree depth, leaf size, and feature subsampling. The same settings were applied in every cross-validation fold and in the final development-partition fit; the untouched test partition was used only for final evaluation.

Test performance was evaluated with R^2^, RMSE, MAE, Pearson r, and mean predicted-minus-actual error residual. Prespecified comparators included a training-mean predictor, linear regression with the same features, a Random Forest using fire-only PM_2·5_ alone, and a Random Forest replacing the fire-only component with the no-fire component. Test-set permutation importance used 10 repeated permutations on a fixed 20,000-row test sample. Accumulated local effects (ALE) estimate how predicted error changes across the observed range of a predictor by averaging local prediction differences; compared with partial dependence, this reduces extrapolation when predictors are correlated. The raw-extreme sensitivity analysis repeated model fitting after restoring the 18 quarantined records. Analyses used Python 3.12.14 with pinned versions of NumPy 2.5.2, pandas 3.0.1, SciPy 1.18.1, scikit-learn 1.9.0, and Matplotlib 3.11.1; full environment and run artifacts accompany the supplement.

## 3. Results

### 3.1 Spatial distribution of wildfire PM_2·5_

CMAQ total PM_2·5_ reflected both anthropogenic and natural sources, with higher long-term concentrations in the San Francisco Bay Area, Central Valley, and Southern California (Fig. 1a). The no-fire simulation retained these urban patterns (Fig. 1b), whereas wildfire-attributable PM_2·5_ was concentrated in northern and forested California, including the Klamath, Six Rivers, and Shasta-Trinity regions (Fig. 1c). Fig. S2 shows strong interannual variation in the wildfire contribution.

### 3.2 Overall and annual model performance

In the primary dataset, mean observed PM_2·5_ was 10.69 μg m^−3^ and mean CMAQ total PM_2·5_ was 9.29 μg m^−3^. Pearson r was 0.502 (r^2^=0.252). CMAQ had a mean bias of − 1.40 μg m^−3^, MAE of 5.16 μg m^−3^, RMSE of 9.96 μg m^−3^, NMB of − 13.09%, and NME of 48.29%. Annual means and bias varied across 2008–2018 (Fig. 2; Table S1), so the aggregate negative bias did not imply uniform performance across years.

### 3.3 Regime-dependent error and threshold detection

Error increased markedly with observed concentration. Background observations accounted for 127,256 pairs and had mean bias − 0.29 μg m^−3^, median error − 1.08 μg m^−3^, MAE 3.43 μg m^−3^, and RMSE 6.07 μg m^−3^. The moderate regime contained 57,644 pairs and had mean bias − 3.54 μg m^−3^, median error − 5.34 μg m^−3^, MAE 7.99 μg m^−3^, and RMSE 11.98 μg m^−3^. Among 2,151 high-regime pairs, mean bias was − 9.79 μg m^−3^, median error was − 17.69 μg m^−3^, MAE was 31.94 μg m^−3^, and RMSE was 50.88 μg m^−3^. CMAQ underestimated 62.8%, 76.0%, and 74.3% of paired observations in the three regimes, respectively (Table S3).

At the 12 μg m^−3^ threshold, 59,795 observed events and 41,071 modeled events produced 27,159 true positives, 32,636 false negatives, and 13,912 false positives. Sensitivity was 0.454, specificity 0.891, precision 0.661, balanced accuracy 0.672, F1 score 0.539, critical success index 0.368, and frequency bias 0.687. CMAQ therefore missed more than half of observed events and underpredicted their frequency.

At 35 μg m^−3^, 2,151 observed events and 3,310 modeled events produced 878 true positives, 1,273 false negatives, and 2,432 false positives. Sensitivity was 0.408, specificity 0.987, precision 0.266, balanced accuracy 0.698, F1 score 0.322, critical success index 0.192, and frequency bias 1.539. Thus, CMAQ generated more high-threshold events overall but often placed them on different monitor-days than the observed events.

Same-day pairing provided the strongest agreement in the whole-day lag sensitivity analysis. Across shifts of one to three days in either direction, Pearson r fell from 0.502 to 0.286–0.420, RMSE rose from 9.96 to 10.55–11.58 μg m^−3^, and F1 score decreased from 0.539 to 0.431–0.495 at 12 μg m^−3^ and from 0.322 to 0.166–0.264 at 35 μg m^−3^. The pooled data therefore do not support a consistent whole-day lag, although event-specific timing errors may still occur (Table S9).

### 3.4 Fire case studies

For the Klamath Fire, the paired dataset contained 958 fire-window observations. CMAQ reproduced much of the temporal variation (r^2^=0.663) but overestimated concentrations on average: observed and modeled means were 18.84 and 27.40 μg m^−3^, mean bias was 8.56 μg m^−3^, NMB was 45.46%, MAE was 12.67 μg m^−3^, and RMSE was 23.33 μg m^−3^ (Fig. 3; Table S2).

For the Rim Fire, 3,611 paired fire-window observations yielded r^2^=0.416. Mean bias was − 0.93 μg m^−3^, NMB was − 11.25%, MAE was 3.88 μg m^−3^, and RMSE was 6.57 μg m^−3^ (Fig. 4; Table S2). The weaker association and negative mean bias were consistent with missed or understated peak concentrations, although the aggregate bias was modest relative to the Klamath result.

### 3.5 Leakage-resistant error diagnostic

The leakage-resistant Random Forest explained 71.1% of variation in CMAQ error in the untouched grouped test partition (Fig. 5a; R^2^=0.711; Pearson r = 0.844; RMSE = 5.82 μg m^−3^; MAE = 3.44 μg m^−3^; n = 37,430). Its mean predicted-minus-actual residual was 0.17 μg m^−3^. Linear regression with the same predictors achieved R^2^=0.600 and RMSE = 6.85 μg m^−3^, while the fire-only Random Forest achieved R^2^=0.592 and RMSE = 6.92 μg m^−3^. Across five grouped cross-validation folds, mean R^2^ was 0.581 (SD = 0.081), mean RMSE was 6.19 μg m^−3^ (SD = 0.63), and mean MAE was 3.41 μg m^−3^ (SD = 0.07), indicating heterogeneity among held-out site-years.

Figure 5a Observed versus predicted CMAQ total PM_2·5_ error for the untouched leakage-resistant test partition. Error was defined as CMAQ total minus observed PM_2·5_. The test partition comprised 37,430 observations from 428 site-year groups that were not used for model fitting or cross-validation. Hexagon color denotes the logarithm of observation count; the dashed line is the 1:1 relation. Axes are limited to the central 99% of plotted values for readability

Post hoc evaluation by observed regime (Fig. 5b) yielded test-set R^2^ values of 0.670 for background, 0.730 for moderate, and 0.695 for high observations. Absolute error nevertheless increased sharply in the high regime: among 473 test observations at or above 35 μg m^−3^, RMSE was 29.23 μg m^−3^ and MAE was 21.06 μg m^−3^. The mean predicted-minus-actual residual was + 16.97 μg m^−3^, indicating that the diagnostic model tended to understate the magnitude of CMAQ underprediction at the highest observed concentrations.

Permutation analysis identified CMAQ fire-only PM_2·5_ as the dominant predictor, followed by longitude, latitude, year, and day of year (Fig. S10). Distance to fire and its missingness indicator contributed little conditional importance. A Random Forest that substituted CMAQ no-fire PM_2·5_ for the fire-only component performed substantially worse (R^2^=0.335). The ALE curves in Figs. S11 and S12 show the direction and nonlinearity of the fitted association between each predictor and CMAQ error while averaging over local changes in the observed data; positive ALE values indicate a more positive predicted CMAQ-minus-observed error relative to the model-wide average. The primary-QC regime error distribution is shown in Fig. S13.

Here, the raw-extreme or upper-tail sensitivity refers specifically to the 18 quarantined CMAQ total PM_2·5_ values above 557 μg m^−3^; it does not refer to every high-regime observation and does not define a general concentration cutoff for model validity. Restoring these records reduced test R^2^ from 0.711 to 0.381 and increased RMSE from 5.82 to 9.44 μg m^−3^. In the full descriptive analysis, restoration increased high-regime RMSE from 50.88 to 150.37 μg m^−3^ and MAE from 31.94 to 38.22 μg m^−3^. These records materially affected squared-error statistics and remain separate from the primary results pending raw-source verification.

## 4. Discussion

The recalculated results show moderate correspondence between CMAQ and observed daily PM_2·5_ across California fire seasons, together with a persistent low bias and substantial paired-event mismatch. The overall correlation (r = 0.502) and mean bias (− 1.40 μg m^−3^) summarize 187,051 site-days, but they obscure the rapid growth in error with observed concentration. RMSE rose from 6.07 μg m^−3^ in the background regime to 50.88 μg m^−3^ in the high regime, and the median high-regime error was − 17.69 μg m^−3^. Aggregate performance should therefore be interpreted alongside concentration-stratified statistics.

Threshold detection adds information that continuous error metrics do not capture. At 12 μg m^−3^, CMAQ underpredicted event frequency and detected only 45.4% of observed events. At the current 35 μg m^−3^ 24-hour standard level, frequency bias exceeded one even though sensitivity was only 40.8% and precision was 26.6%. This combination means that the model generated too many high-level events in aggregate while failing to align most of them with observed high-concentration site-days. These paired daily classifications do not themselves determine regulatory attainment, which uses a multi-year design value, but the mismatch is relevant to health and policy applications of wildfire-smoke estimates [19].

The two fire case studies illustrate event heterogeneity. Klamath showed stronger temporal correspondence but a positive mean bias of 8.56 μg m^−3^, whereas Rim showed weaker correspondence and a mean bias of − 0.93 μg m^−3^. The available data do not isolate a single cause. Plausible contributors include event-specific uncertainty in emissions, plume injection, transport, and sub-grid concentration gradients [20, 21].

The leakage-resistant diagnostic addresses a separate question: whether wildfire loading and spatial-temporal context explain CMAQ error without giving the model observed PM_2·5_, CMAQ total PM_2·5_, or a combination of components that reconstructs total PM_2·5_. The grouped test R^2^ of 0.711 and mean cross-validation R^2^ of 0.581 show that the signal persists after those leakage routes are removed and after site-year grouping limits dependence between development and evaluation records. Fire-only CMAQ PM_2·5_ provided most of the predictive information, while geographic and temporal variables contributed smaller increments.

The Random Forest should be interpreted as a diagnostic, not an operational bias-correction model. Its validation remained internal to the available California record, fold performance varied among site-years, and absolute error was large for high observations despite a relatively high within-regime R^2^. The positive residual of 16.97 μg m^−3^ in the high test regime shows that the fitted diagnostic understated the magnitude of CMAQ underprediction in those cases. It cannot determine what fraction of conventional threshold classifications are “real” or supply validated corrected counts for the three concentration regimes. External validation across other regions, CMAQ configurations, emissions inventories, and fire characteristics would be required before deployment or before recommending model settings by fire type.

The extreme-value sensitivity analysis also limits inference about the 18 quarantined values. Their restoration substantially degraded squared-error metrics, but the paired dataset alone cannot establish whether they are physical extremes, extraction artifacts, or processing errors. This result does not identify a PM_2·5_ concentration above which CMAQ output should be treated as invalid. Quarantining the values without capping preserves the primary analysis and a transparent sensitivity result; verification against the original gridded CMAQ output remains necessary before the records can be classified.

Taken together, the findings support CMAQ for regional source-attribution and multi-year exposure analyses, particularly where total and no-fire simulations are used to estimate wildfire contributions. They also identify settings that require caution: localized exposure estimation, event-day classification, and extreme smoke concentrations. A broader evaluation across the contiguous United States, stratified by prescribed versus wildfire emissions, fire size, fuel type, duration, and model configuration, would be needed to determine how these errors affect health analyses or whether settings should vary by fire type. For current applications, model output should be evaluated with paired threshold metrics and, where possible, combined with monitoring, satellite, or observation-fusion products.

## 5. Conclusions

Across California fire seasons from 2008–2018, CMAQ captured part of the daily variability in observed PM_2·5_ but underestimated concentrations on average and showed increasingly large error at higher observed concentrations. The key event-detection result was not simply underprediction: CMAQ missed more than half of observed events at both 12 and 35 μg m^−3^, and at 35 μg m^−3^ it simultaneously overpredicted total event frequency while poorly matching the site-days on which observed high concentrations occurred. Shifting modeled and observed series by one to three days did not improve pooled agreement, providing no evidence of a consistent whole-day lag across sites and years.

A leakage-resistant Random Forest confirmed that CMAQ error contains structured information associated mainly with modeled wildfire PM_2·5_ and, to a lesser extent, spatial and temporal context. Grouped validation produced an untouched-test R^2^ of 0.711, but high-concentration absolute errors remained large. The diagnostic is useful for understanding model behavior, not yet for operational correction.

CMAQ remains suitable for regional and multi-year assessments when uncertainty is reported explicitly. Studies focused on individual events or localized extreme exposure should use concentration-stratified and paired threshold-detection diagnostics, preserve the distinction between primary and raw-extreme sensitivity results, and verify the quarantined upper-tail records before incorporating them into inference.

## Supplementary Material

This is a list of supplementary files associated with this preprint. Click to download.

- CMAQWildfirePM25SupplementDiscoverEnvironmentSubmissionFinal.docx

## Figures and Tables

**Figure 1:**
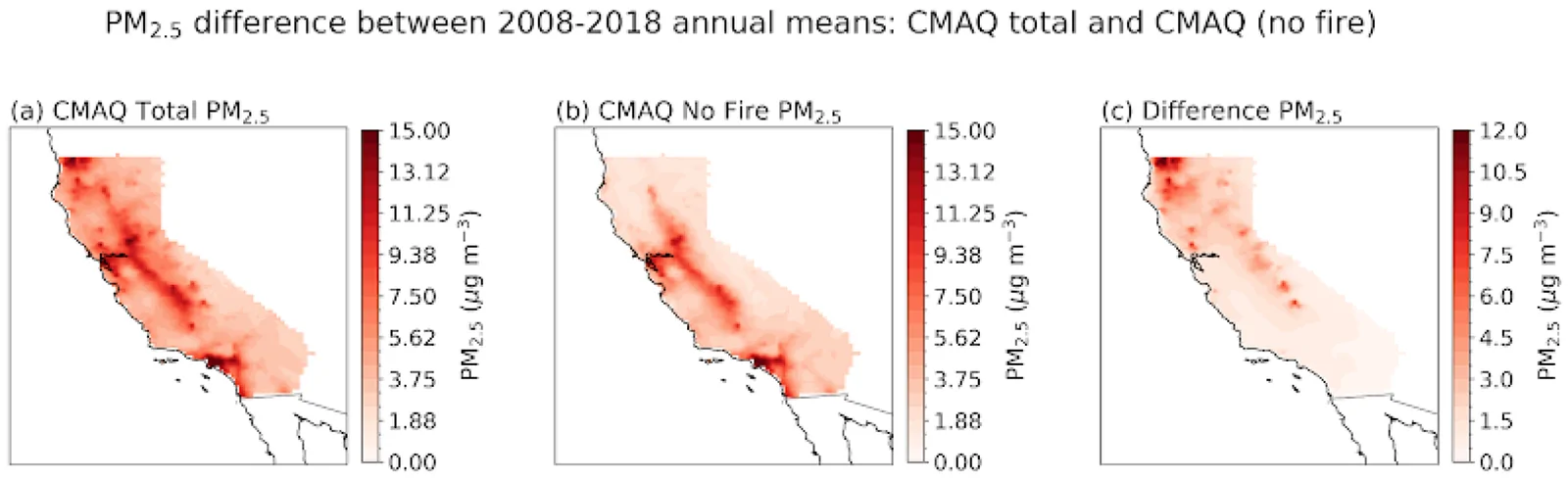
Spatial distribution of annual mean PM_2·5_ concentrations across California for 2008–2018 from CMAQ simulations. Panel (a) shows total PM_2·5_ from all emission sources, panel (b) shows PM_2·5_ from the no-fire simulation (excluding wildfire emissions), and panel (c) shows wildfire-attributable PM_2·5_ calculated as the difference between total and no-fire simulations. All concentrations are reported in μg m^−3^

**Figure 2:**
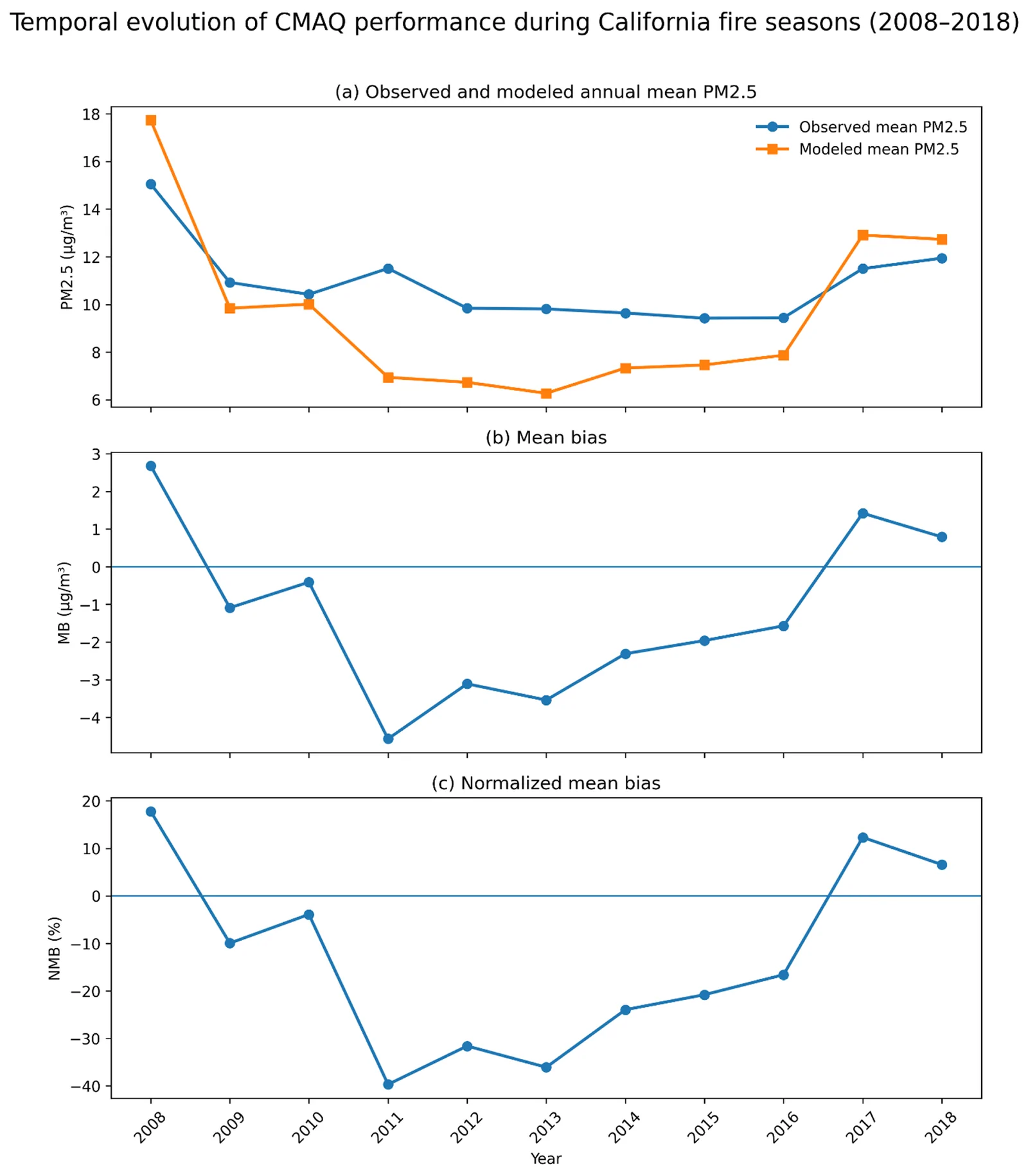
Temporal evolution of CMAQ performance during California fire seasons (June–October), 2008–2018. Panel (a) compares observed and modeled year-specific fire-season mean PM_2·5_ concentrations, illustrating interannual variability and systematic differences in magnitude. Panel (b) shows annual mean bias (MB), and panel (c) shows annual normalized mean bias (NMB), highlighting temporal shifts between overestimation and underestimation across the study period. Positive values indicate model overestimation, whereas negative values indicate model underestimation

**Figure 3:**
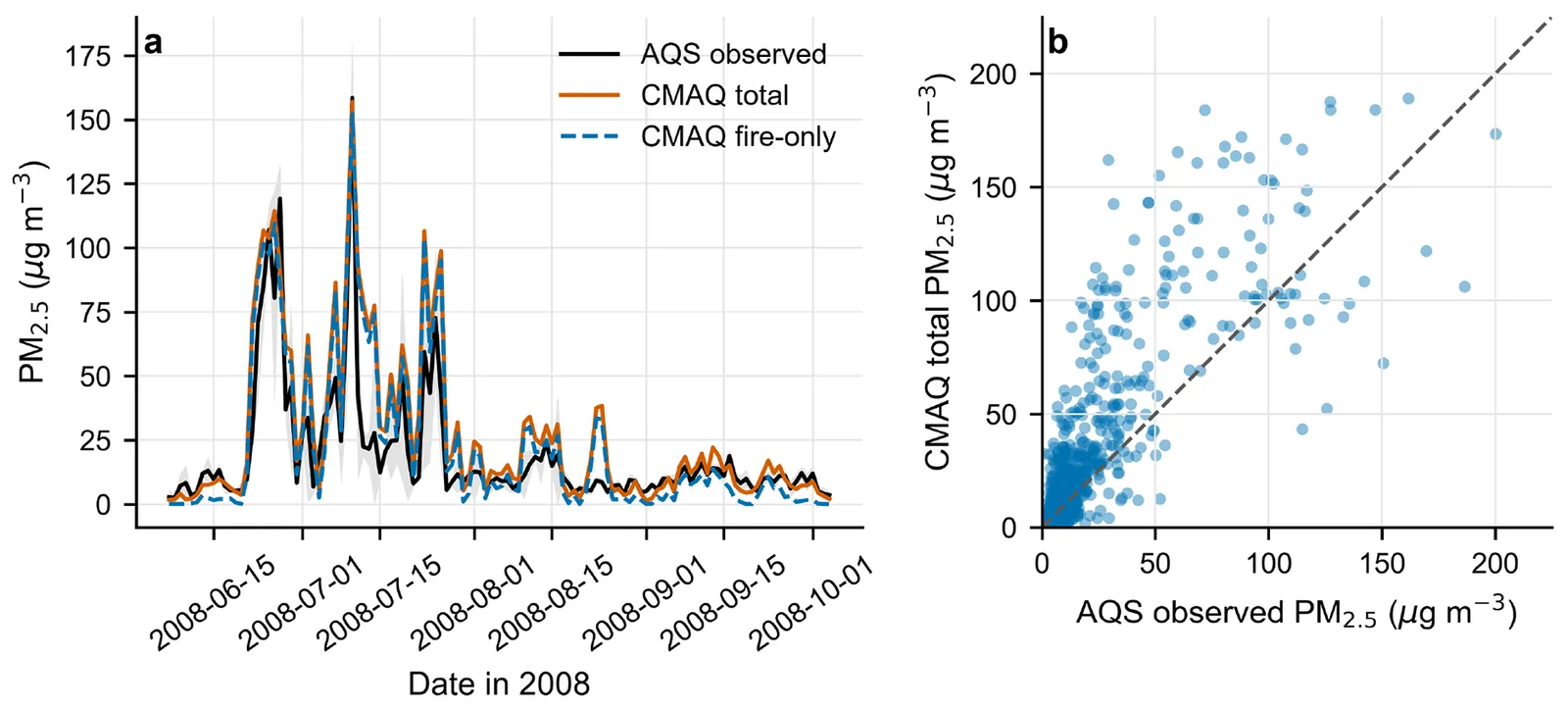
Daily and paired-site-day evaluation for the 2008 Klamath Fire window. a Daily across-site mean AQS-observed PM_2·5_ (black), CMAQ total PM_2·5_ (orange), and CMAQ fire-only PM_2·5_ (blue dashed) for monitors within 200 km of the recorded fire origin; shading shows ±1 standard deviation of observed concentrations. b Paired AQS observations and CMAQ total PM_2·5_ for the 958 retained monitor-days; the dashed line shows the 1:1 relation. Concentrations are in μg m^−3^

**Figure 4:**
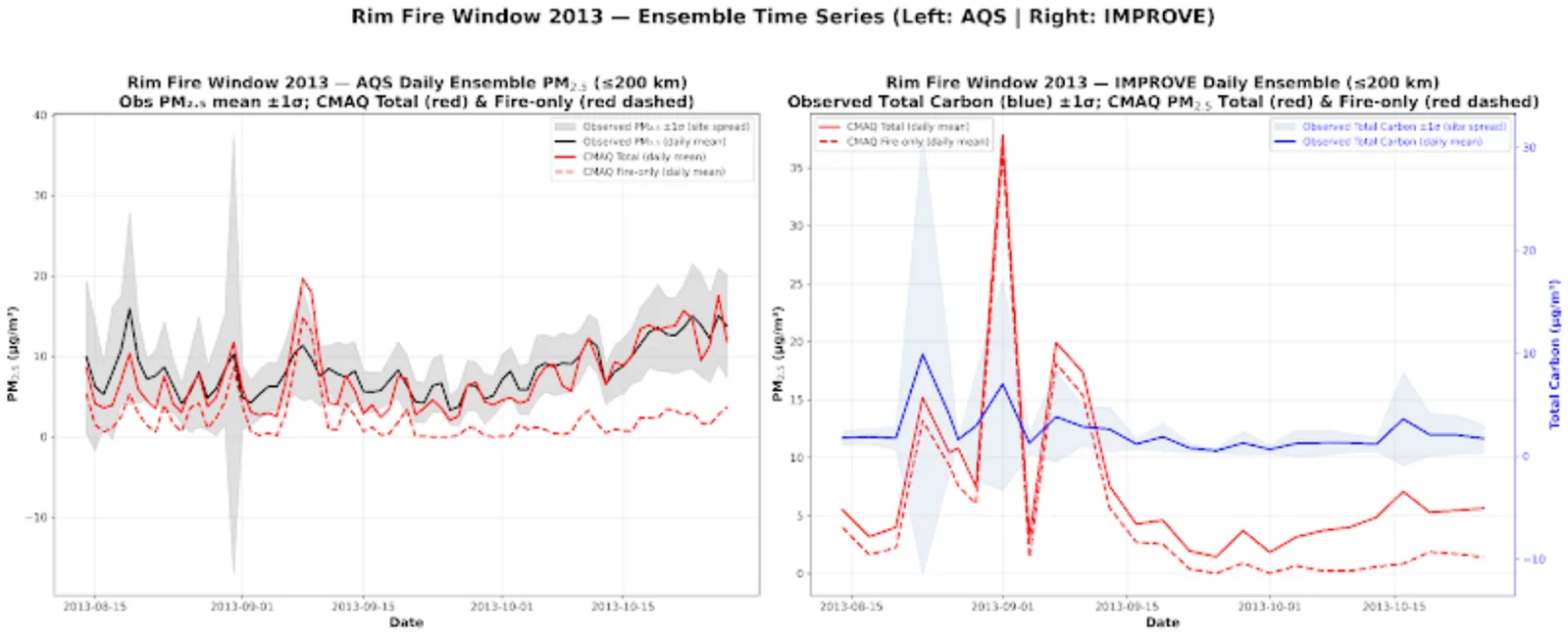
Time series of PM_2·5_ concentrations during the 2013 Rim Fire comparing observations and CMAQ simulations. The left panel shows daily PM_2·5_ from AQS observations (black line) and CMAQ estimates within a 200 km radius, including total PM_2·5_ (solid red line) and fire-only PM_2·5_ (dashed red line). The right panel shows observed elemental carbon (EC) concentrations from IMPROVE sites (blue line) alongside CMAQ estimates of total and fire-only PM_2·5_. Shaded regions represent the standard deviation of observations (gray for AQS and blue for IMPROVE). The analysis period spans August 14 to October 27, 2013. All concentrations are reported in μg m^−3^

**Figure 5:**
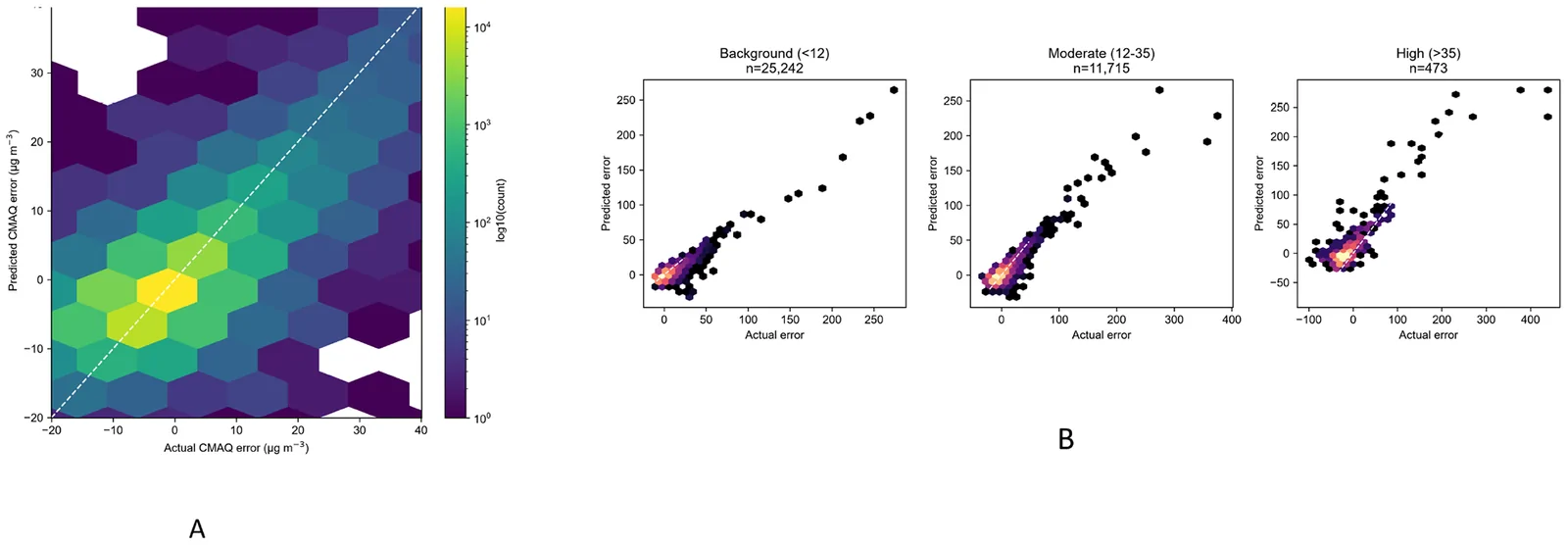
a Observed versus predicted CMAQ total PM_2·5_ error for the untouched leakage-resistant test partition. Error was defined as CMAQ total minus observed PM_2·5_. The test partition comprised 37,430 observations from 428 site-year groups that were not used for model fitting or cross-validation. Hexagon color denotes the logarithm of observation count; the dashed line is the 1:1 relation. Axes are limited to the central 99% of plotted values for readability b Observed versus predicted CMAQ total PM_2·5_ error in the untouched test partition, stratified post hoc by observed PM_2·5_ concentration regime. Regime labels were not supplied to the model. Panels show background (<12 μg m^−3^), moderate (12 to <35 μg m^−3^), and high (≥35 μg m^−3^) observations. Hexagon color denotes the logarithm of observation count; dashed lines are the 1:1 relation

## Data Availability

The data and code required to reproduce the reported analyses are available in the frozen reproducibility package archived in Zenodo (version 1.0.0; https://doi.org/10.5281/zenodo.22677978). The corresponding public GitHub repository is available at https://github.com/boxeman/cmaq-wildfire-pm25-california.
